# Sex-Based Differences in Clinical Outcomes With Edoxaban Therapy: A Prespecified Analysis of the EPIC-CAD Trial

**DOI:** 10.1016/j.jacasi.2025.12.022

**Published:** 2026-02-28

**Authors:** Gi-Hwan Kim, MinSoo Cho, Seonok Kim, Do-Yoon Kang, Jung-Min Ahn, Yong-Seog Oh, Chang Hoon Lee, Eue-Keun Choi, Ji Hyun Lee, Chang Hee Kwon, Gyung-Min Park, Hyung Oh Choi, Kyung-Ha Park, Kyoung-Min Park, Jongmin Hwang, Ki-Dong Yoo, Young-Rak Cho, Ji Hyun Kim, Ki Won Hwang, Eun-Sun Jin, Osung Kwon, Ki-Hun Kim, Seung-Jung Park, Gi-Byoung Nam, Duk-Woo Park, EPIC-CAD Investigators

**Affiliations:** a Department of Cardiology, Asan Medical Center, University of Ulsan College of Medicine, Seoul, Korea; b Division of Biostatistics, Asan Medical Center, University of Ulsan College of Medicine, Seoul, Korea; c Department of Cardiology, Seoul St Mary’s Hospital, Seoul, Korea; d Department of Cardiology, Veterans Health Service Medical Center, Seoul, Korea; e Cardiovascular Center, Seoul National University Bundang Hospital, Seongnam, Korea; f Division of Cardiology, Konkuk University Medical Center, Seoul, Korea; g Department of Cardiology, Ulsan University Hospital, Ulsan, Korea; h Department of Cardiology, Soon Chung Hyang University Hospital Bucheon, Bucheon, Korea; i Department of Cardiology, Hallym University Medical Center, Anyang, Korea; j Division of Cardiology, Samsung Medical Center, Seoul, Korea; k Department of Cardiology, Keimyung University Dongsan Hospital, Daegu, Korea; l Department of Cardiology, St Vincent’s Hospital, Suwon, Korea; m Department of Cardiology, Dong-A University Hospital, Busan, Korea; n Department of Cardiology, Dongguk University Ilsan Hospital, Goyang, Korea; o Department of Cardiology, Pusan National University Yangsan Hospital, Yangsan, Korea; p Department of Cardiology, Kyung Hee University Hospital at Gangdong, Seoul, Korea; q Division of Cardiology, Eunpyeong St Mary’s Hospital, Seoul, Korea; r Department of Cardiology, Haeundae Paik Hospital, Busan, Korea

**Keywords:** anticoagulation, atrial fibrillation, coronary artery disease, sex difference

## Abstract

**Background:**

Sex-based differences exist in the characteristics and prognosis of patients with atrial fibrillation (AF) and coronary artery disease (CAD). It is still unknown whether optimal antithrombotic therapy differs by sex.

**Objectives:**

This study aimed to evaluate the efficacy and safety of edoxaban therapy in AF with stable CAD, according to sex category.

**Methods:**

This prespecified substudy of the EPIC-CAD (Edoxaban Versus Edoxaban With Antiplatelet Agent in Patients With Atrial Fibrillation and Chronic Stable Coronary Artery Disease [EPIC-CAD]) trial compared edoxaban monotherapy with dual antithrombotic therapy (edoxaban plus a single antiplatelet agent). The primary outcome was net adverse clinical events (a composite of death, myocardial infarction, stroke, unplanned revascularization, or clinically relevant bleeding) at 12 months. Median follow-up was 12.0 months (IQR: 11.6-12.9).

**Results:**

Of 1,040 randomized patients, 238 (22.9%) were women; in the dual-therapy group (n = 516), 110 (21.3%) were women and in the monotherapy group (n = 524), 128 (24.4%) were women. Women were older, had lower body weight, lower creatinine clearance, and more frequently met the edoxaban dose-reduction criteria. The risk of primary outcome was similar between women and men (adjusted HR [aHR]: 1.15; 95% CI: 0.71-1.87; *P* = 0.559). Edoxaban monotherapy significantly reduced primary events in men compared with dual therapy (aHR: 0.36; 95% CI: 0.23-0.57; *P* < 0.001), but not in women (aHR: 0.74; 95% CI: 0.33-1.64; *P* = 0.455). No significant interaction between the randomized treatment and sex was observed (*P* for interaction = 0.09).

**Conclusions:**

Edoxaban monotherapy reduced primary net adverse events at 12 months in men but not in women. No significant interaction with sex was observed. Further research is needed to identify sex-specific antithrombotic strategies for patients with AF and CAD. (Edoxaban Versus Edoxaban With Antiplatelet Agent in Patients With Atrial Fibrillation and Chronic Stable Coronary Artery Disease [EPIC-CAD]; NCT03718559)

Several studies have reported notable sex differences in the epidemiology and clinical management of atrial fibrillation (AF).^1^, ^2^, ^3^ Women tend to be older and more symptomatic than men, have a higher risk of stroke and bleeding events, and experience differences in prognosis despite receiving oral anticoagulant (OAC) therapy. The pathophysiology, clinical presentation, and prognosis of atherosclerotic coronary artery disease (CAD) also vary significantly between sexes.^4^, ^5^, ^6^, ^7^ Moreover, substantial sex-related differences exist in the extent of atherosclerotic disease burden, patterns of medical therapy and coronary revascularization, and their efficacy and safety.

Given that AF is the most prevalent cardiac arrhythmia and atherosclerotic CAD is the most common cardiovascular disease,^8^^,^^9^ their coexistence is common; however, selecting optimal antithrombotic therapy for patients with both conditions remains challenging.^10^^,^^11^ In these patients, the combined use of anticoagulants and antiplatelet therapy significantly increases the risk of bleeding.^12^^,^^13^ Moreover, it remains uncertain whether the optimal antithrombotic strategy for high-risk patients with both AF and CAD should differ based on sex. If sex-specific disparities exist, physicians may need to consider personalized antithrombotic strategies to balance the risks of ischemia and bleeding.

The EPIC-CAD (Edoxaban Versus Edoxaban With Antiplatelet Agent in Patients With Atrial Fibrillation and Chronic Stable Coronary Artery Disease [EPIC-CAD]) trial was a randomized trial comparing edoxaban monotherapy with dual antithrombotic therapy (edoxaban plus a single antiplatelet agent) in patients with AF and stable CAD.^14^ Given sex-specific disparities in clinical characteristics and differences in susceptibility to ischemic and bleeding risks with antithrombotic therapy, this prespecified analysis of EPIC-CAD data aims to enhance the interpretation of the trial’s findings by sex. Therefore, we examined clinical outcomes based on the randomized antithrombotic strategy (edoxaban monotherapy vs dual antithrombotic therapy) and sex in patients with concomitant AF and CAD.

## Methods

### Study population

The design, methods, and primary findings of the EPIC-CAD trial have been previously reported.^14^ In brief, the EPIC-CAD trial was a multicenter, open-label, adjudicator-masked, clinical trial conducted at 18 hospitals across South Korea from May 2019 to September 2022. A total of 1,040 patients with high-risk AF (CHA_2_DS_2_-VASc score ≥2) and stable CAD (either revascularized or managed medically) were randomly assigned in a 1:1 ratio to receive either edoxaban monotherapy or dual antithrombotic therapy (edoxaban plus a single antiplatelet agent). Stable CAD was defined as chronic coronary syndrome previously treated with percutaneous coronary intervention or coronary artery bypass grafting for at least 6 months, acute coronary syndrome previously treated with percutaneous coronary intervention or coronary artery bypass grafting for at least 12 months before enrollment, or anatomically confirmed obstructive CAD (≥50% stenosis of a major epicardial coronary artery) managed with medical therapy alone. Key exclusion criteria included contraindications to antithrombotic drugs, severe comorbidities associated with a high risk of bleeding, a history of intracranial hemorrhage, mechanical prosthetic valve, or significant mitral stenosis.

The study protocol was approved by the institutional review board or ethics committee of each participating center. All patients provided written informed consent before enrollment. For this prespecified secondary analysis, the study participants were categorized according to their sex (women or men). Sex-related data of the participants were self-reported in the EPIC-CAD trial and collected from medical records.

Enrolled patients were randomly assigned to receive either standard-dose edoxaban monotherapy (60 mg once daily) or dual antithrombotic therapy, including standard-dose edoxaban plus a single antiplatelet agent (either aspirin or a P2Y12 inhibitor, at the discretion of the treating physician) for 12 months. A reduced dose of edoxaban (30 mg once daily) was prescribed for patients meeting any of the following dose-reduction criteria: 1) body weight ≤60 kg; 2) moderate-to-severe renal impairment (calculated creatinine clearance of 15-50 mL/min); or 3) concomitant use of P-glycoprotein inhibitors (cyclosporine, dronedarone, erythromycin, or ketoconazole). Among P2Y12 inhibitors, the use of clopidogrel was preferred.

### Study data adjudication and outcome definitions

The primary outcome of the study was net adverse clinical events (ie, efficacy and safety outcomes), defined as a composite of death from any cause, myocardial infarction (MI), stroke, systemic embolism, unplanned urgent revascularization, or major bleeding or clinically relevant nonmajor bleeding (as defined by the International Society on Thrombosis and Haemostasis)^15^ at 12 months after randomization. Secondary efficacy outcomes included the individual components of the primary outcome, a composite of any ischemic event (death from any cause, MI, ischemic stroke, systemic embolism, or unplanned urgent revascularization), and a composite of major ischemic events (death from any cause, MI, ischemic stroke, or systemic embolism). Secondary safety outcomes included a composite of major bleeding or clinically relevant nonmajor bleeding, major bleeding, clinically relevant nonmajor bleeding, and any bleeding event. Prespecified, standard definitions were used for the assessment of clinical outcomes.^14^ All clinical outcomes were adjudicated by an independent clinical events committee, whose members were unaware of the trial-group assignments.

Follow-up assessments were performed at baseline and 6 and 12 months after randomization. At each visit, data on clinical events and concomitant cardiovascular medications were systematically collected. Cross-validation of survival status was performed using the Korean National Health Insurance database.

### Statistical analysis

The full statistical analysis plan, including the sample size calculation for the EPIC-CAD trial, has been previously published.^14^ All analyses were conducted on an intention-to-treat basis. Baseline characteristics between sexes or randomized strategies were compared using the chi-square or Fisher exact test for categorical variables and the Kruskal-Wallis test for continuous variables. Time-to-event estimates for clinical outcomes were obtained by use of Kaplan-Meier estimates and compared with the log-rank test.

For sex-based comparison (women vs men), the propensity score was estimated using a multivariable logistic regression model, which included age, body mass index, hypertension, diabetes, cerebrovascular accident history, CHA_2_DS_2_-VASc score, HAS-BLED score, prior revascularization, and the indication for edoxaban dose adjustment. Given the approximately 2:8 sex distribution, 1:4 nearest-neighbor matching without replacement was performed with a caliper width of 0.2 of the standard deviation of the logit of the propensity score. The quality of matching was evaluated by assessing covariate balance between groups using a standardized mean difference of <0.1 to indicate adequate balance. After propensity score matching, regression analyses accounted for the matched design. Matched pairs were treated as strata, and conditional logistic regression was used for binary outcomes. For continuous outcomes, linear mixed-effects models with a random intercept for each matched set were applied to account for within-pair correlation.

To compare the effects of edoxaban monotherapy vs dual antithrombotic therapy in each arm of women and men, treatment effects were estimated with Cox proportional hazards regression and are presented as HRs with 95% CIs. Unadjusted analyses were performed first, followed by multivariable Cox regression to adjust for confounders including age, BMI, CHA_2_DS_2_-VASc score, HAS-BLED score, prior coronary revascularization, and edoxaban dose-reduction criteria. The proportional hazards assumption was tested using Schoenfeld residuals and visual inspection. Interactions between randomized antithrombotic strategy and sex were also assessed.

Missing data for baseline variables were minimal, with no evidence of systematic patterns. Although formal testing for missing completely at random was not performed, the missingness appeared random; therefore, complete case analyses were conducted without imputation. Subgroup analyses of the primary outcome were performed in each cohort of women and men, based on key clinical factors. All reported *P* values are 2 sided. A *P* value of <0.05 was considered statistically significant for all tests. No adjustments were made for multiple testing; therefore, all findings of the present study must be interpreted as exploratory, given the potential for type I error owing to multiple comparisons. All statistical analyses were performed by independent statisticians using commercially available software (R software version 4.6.1).

## Results

### Baseline characteristics of the study cohort

**Figure 1:**
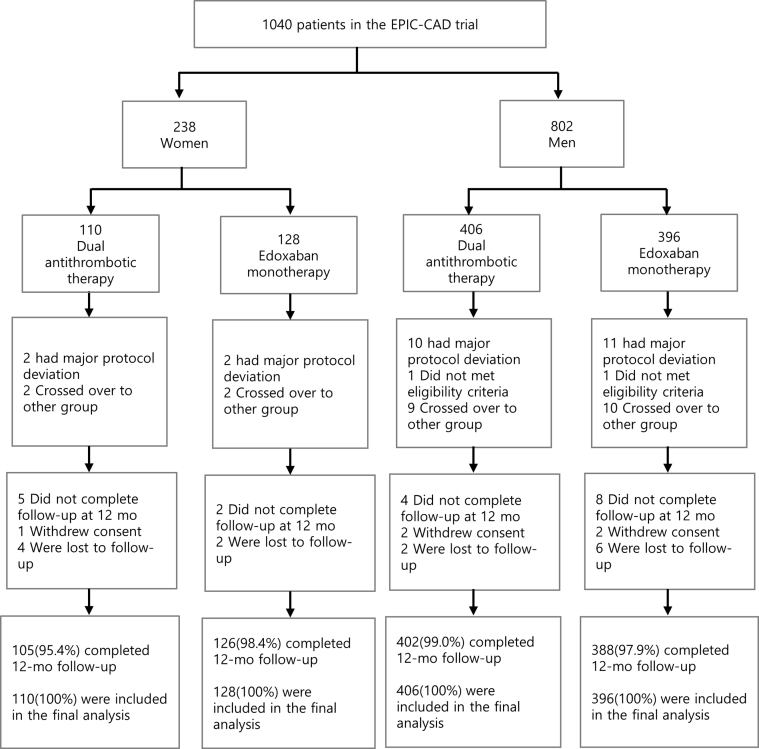
Patient Enrollment, Randomization, and Follow-Up by Sex A study flow diagram of patients stratified by edoxaban monotherapy and dual antithrombotic therapy is provided. Only patients who survived, did not withdraw, and were not lost to follow-up at 12 months post randomization were included. Percentages may not total 100% due to rounding. EPIC-CAD = Edoxaban Versus Edoxaban With Antiplatelet Agent in Patients With Atrial Fibrillation and Chronic Stable Coronary Artery Disease.

Between May 14, 2019, and September 19, 2022, a total of 1,040 patients were enrolled in the EPIC-CAD trial at 18 sites across South Korea. Among them, 238 (22.9%) were women and 802 (77.1%) were men. Of the 238 women, 128 (53.8%) were randomized to receive edoxaban monotherapy and 110 (46.2%) received dual antithrombotic therapy (Figure 1). Among the 802 men, 396 (49.4%) were randomized to receive edoxaban monotherapy and 406 (50.6%) received dual antithrombotic therapy.

Baseline characteristics for women and men are summarized in Table 1. Compared with men, women were older, had lower body weight and creatinine clearance, and had higher CHA_2_DS_2_-VASc and CHA_2_DS_2_ scores. Also, the proportion of women meeting the criteria for edoxaban dose-reduction—because of factors such as lower renal function and lower body weight—was approximately twice that of men. There was no significant difference between the sexes in bleeding risk, including the HAS-BLED score, or in rhythm control strategies such as radiofrequency catheter ablation.

**Table 1:** Baseline Characteristics by Sex

	Women (n = 238)	Men (n = 802)	*P* Value
Age, y	75.2 ± 7.4	71.1 ± 8.2	<0.001
Weight, kg	59.6 ± 9.7	71.4 ± 10.3	<0.001
Body mass index, kg/m^2^	25.5 ± 3.8	25.3 ± 3.2	0.466
Cardiac risk factors and comorbidities			
Diabetes mellitus	97 (40.8)	324 (40.4)	0.981
Hypertension	195 (81.9)	650 (81.0)	0.832
Hyperlipidemia or statin use	227 (95.4)	745 (92.9)	0.225
Current smoker	6 (2.5)	81 (10.1)	<0.001
Previous MI	30 (12.6)	141 (17.6)	0.086
Congestive heart failure	48 (20.2)	157 (19.6)	0.913
History of cerebrovascular disease	42 (17.6)	112 (14.0)	0.193
History of peripheral artery disease	15 (6.3)	63 (7.9)	0.510
Creatinine clearance, mL/min	57.7 ± 21.3	69.2 ± 22.3	<0.001
Type of atrial fibrillation			0.077
Paroxysmal	144 (60.5)	431 (53.7)	
Persistent or permanent	94 (39.5)	371 (46.3)	
CHA_2_DS_2_-VASc score			<0.001
Mean	5.2 ± 1.6	4.1 ± 1.4	
Median (IQR)	5 (4-6)	4 (3-5)	
CHA_2_DS_2_ score			0.002
Mean	2.3 ± 1.3	2.1 ± 1.2	
Median (IQR)	2 (2-3)	2 (1-3)	
HAS-BLED score			0.097
Mean	2.2 ± 0.8	2.1 ± 0.8	
Median (IQR)	2 (2-3)	2 (2-3)	
Obstructive CAD managed medically	93 (39.1)	264 (32.9)	0.158
Previous coronary revascularization	145 (60.9)	538 (67.1)	0.093
Previous PCI	131 (55.0)	495 (61.7)	0.076
Drug-eluting stent, n/N (%)	110 (84.0)	408 (82.4)	0.775
Bare-metal stent, n/N (%)	3 (2.3)	17 (3.4)	0.702
Both stent type, n/N (%)	2 (1.5)	10 (2.0)	0.994
Unknown stent type, n/N (%)	16 (12.2)	60 (12.1)	1.000
Previous CABG	21 (8.8)	56 (7.0)	0.417
Previous rhythm control strategy			
Previous RFCA	70 (29.4)	212 (26.4)	0.410
Previous or concomitant PPI use	31 (13.0)	102 (12.7)	0.989
Indication for dose adjustment of edoxaban	147 (61.8)	199 (24.8)	<0.001
Edoxaban dose			<0.001
60 mg/d	80 (33.6)	518 (64.6)	
30 mg/d	158 (66.4)	284 (35.4)	
Antiplatelet used	108 (45.4)	407 (50.7)	0.110
Aspirin	76 (31.9)	244 (30.4)	0.549
Clopidogrel	32 (14.3)	163 (20.3)	0.182

Values are mean ± SD or n (%) unless otherwise stated. Percentages may not sum to 100% due to rounding.

CABG = coronary artery bypass grafting; CAD = coronary artery disease; MI = myocardial infarction; PCI = percutaneous coronary intervention; PPI = proton pump inhibitor; RFCA = radiofrequency catheter ablation.

The baseline characteristics of patients randomized to edoxaban monotherapy vs dual antithrombotic therapy stratified by sex are summarized in Supplemental Table 1. Sex differences were observed in the frequency of edoxaban underdosing in both the dual antithrombotic therapy and monotherapy groups. In both groups, women were more likely to receive 30 mg of edoxaban than men, with the odds of women receiving the lower dose being twice as high as for men. On the other hand, most baseline characteristics, stratified by the randomized antithrombotic strategy for each cohort of women and men, did not differ significantly.

### Sex differences in event rates

**Figure 2:**
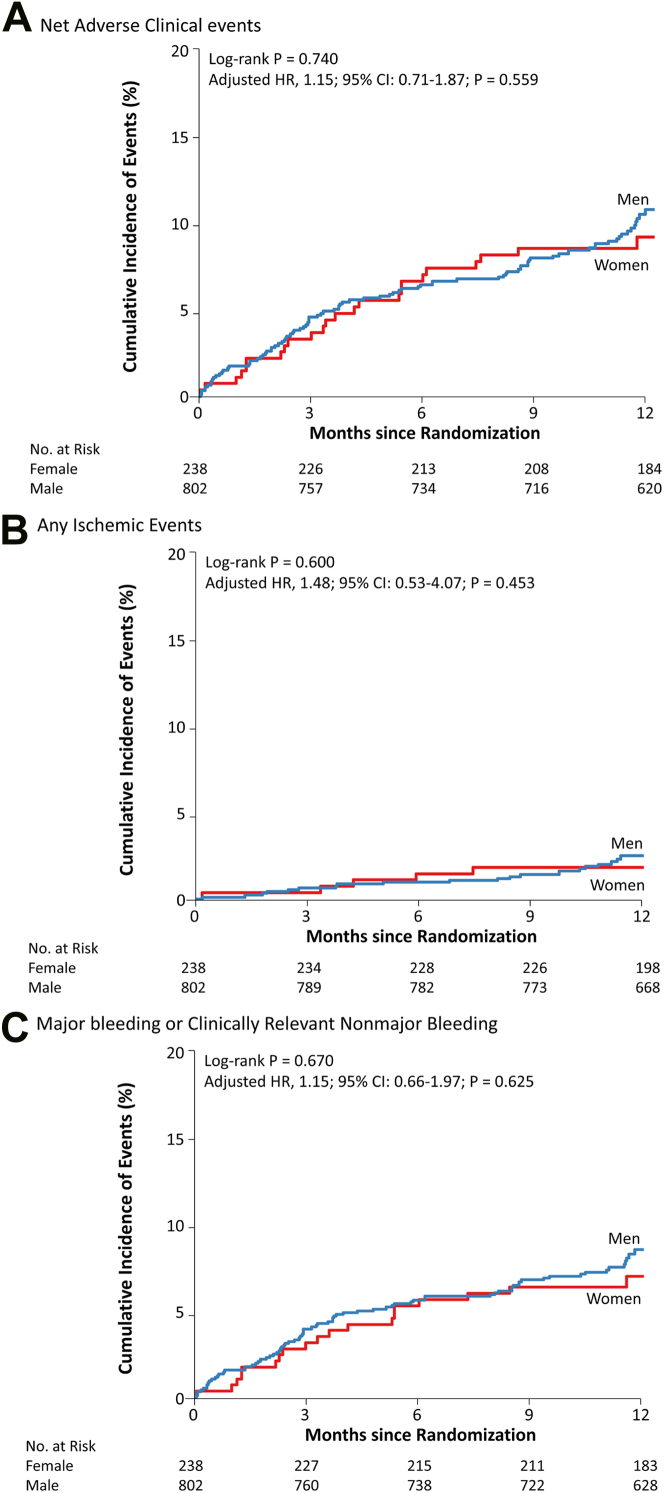
Kaplan-Meier Curves by Sex for Primary and Secondary Outcomes Primary outcome refers to the net adverse outcome, which includes death from any cause, myocardial infarction, stroke, systemic embolism, unplanned procedures, and major or clinically relevant nonmajor bleeding. Secondary outcomes included major ischemic events and clinically relevant bleeding. Kaplan-Meier curves show the cumulative incidence of the primary outcome over 12 months, stratified by sex. Statistical significance was assessed using multivariable Cox regression analyses, adjusted for clinically relevant covariates, including age, body mass index, CHA_2_DS_2_-VASc score, HAS-BLED score, history of prior coronary revascularization, and the presence of edoxaban dose-reduction criteria.

The median follow-up was 12.0 months (IQR: 11.6-12.9) and was similar between women (12.0 months, IQR: 11.6-13.1) and men (12.0 months, IQR: 11.7-12.9). Of the overall cohort (n = 1,040), 1,021 patients (98.2%) completed primary and secondary outcome assessments at 12 months, including 231 of 238 women (97.1%) and 790 of 802 men (98.5%) (Figure 1). Data regarding the vital statuses were obtained for all patients. The primary and secondary clinical outcomes between women and men are summarized in Supplemental Table 2. The incidence of the primary net adverse event at 12 months was similar between women and men (10.6% vs 11.8%, respectively; adjusted HR: 1.15; 95% CI: 0.71-1.87; *P* = 0.559) (Figure 2). The cumulative incidence of individual components of the primary outcome also appeared to be similar. The cumulative incidence of any ischemic event (2.1% vs 2.6%, respectively; adjusted HR: 1.48; 95% CI: 0.53-4.07; *P* = 0.453) and major ischemic events (2.1% vs 1.5%, respectively; adjusted HR: 0.83; 95% CI: 0.27-2.58; *P* = 0.752) at 12 months did not differ significantly between sexes. The estimated cumulative incidence of major bleeding or clinically relevant nonmajor bleeding at 12 months was also similar between women and men (8.2% vs 9.5%, respectively; adjusted HR: 1.15; 95% CI: 0.66-1.97; *P* = 0.625).

### Sex differences in event rates with edoxaban therapy

**Figure 3:**
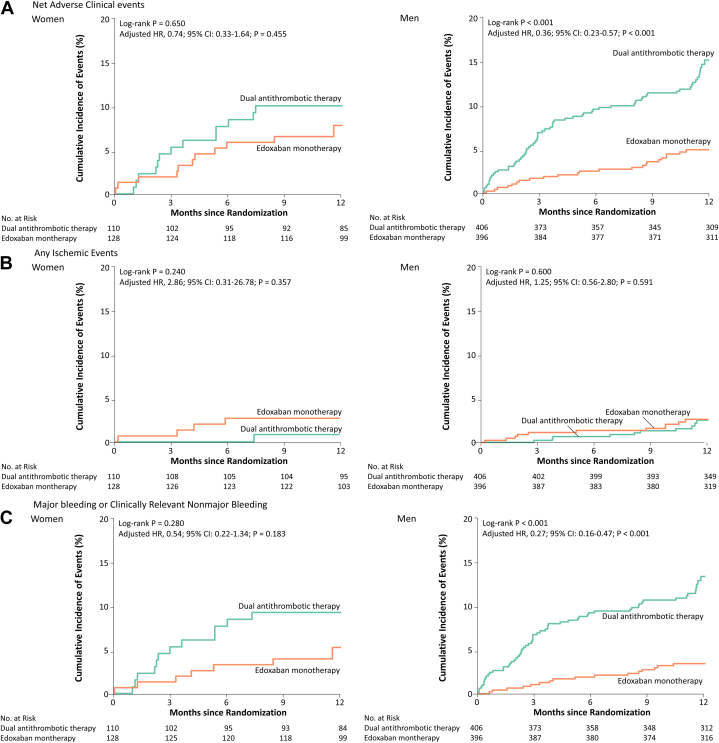
Kaplan-Meier Analysis of Outcomes by Sex and Edoxaban Therapy This figure compares the primary endpoint (net adverse outcomes) between edoxaban monotherapy and dual antithrombotic therapy, as well as secondary outcomes, including ischemic events and major bleeding, in both women and men, using Kaplan-Meier curves. Statistical significance was assessed using multivariable Cox regression analyses, as detailed in Figure 2.

The primary and secondary outcomes according to the randomized antithrombotic strategy, stratified by sex, are presented in Table 2. Among women, when the outcomes were compared based on the randomized antithrombotic regimen, the incidence of the primary net adverse event at 12 months was not significantly different between edoxaban monotherapy and dual antithrombotic therapy (9.4% vs 12.1%, respectively; adjusted HR: 0.74; 95% CI: 0.33-1.64; *P* = 0.455) (Figure 3). However, in men, the incidence of the primary net adverse event at 12 months was significantly lower with edoxaban monotherapy than with dual antithrombotic therapy (6.0% vs 17.4%; adjusted HR: 0.36; 95% CI: 0.23-0.57; *P* < 0.001). Given the trend toward a lower incidence of the primary outcome with edoxaban monotherapy in both sexes, there was no significant interaction between the randomized antithrombotic strategy and sex category (*P* for interaction = 0.092).

**Table 2:** 12-Month Clinical Outcomes by Randomized Antithrombotic Strategy in Women and Men

					*P* Value	*P* Value for Interaction
	Dual Antithrombotic Therapy	Edoxaban Monotherapy	Unadjusted HR (95% CI)	Adjusted HR (95% CI)		
Women	n = 110	n = 128				
Primary outcome						
Net adverse clinical events	13 (12.1)	11 (9.4)	0.81 (0.39-1.72)	0.74 (0.33-1.64)	0.455	0.092
Secondary outcomes						
Efficacy outcomes						
Death	0 (0.0)	2 (1.6)	2.43 (0.20-29.23)	NR	0.795	0.510
Cardiovascular cause	0 (0.0)	0 (0.0)	3.52 (0.16-75.51)	NR	1.000	0.397
Noncardiovascular cause	0 (0.0)	2 (1.6)	0.81 (0.01-100.47)	NR	NR	0.913
Stroke	1 (1.0)	3 (2.4)	2.02 (0.26-15.91)	2.89 (0.29-29.17)	0.369	0.859
Ischemic event	1 (1.0)	2 (1.6)	1.44 (0.15-13.48)	NR	1.000	0.866
Hemorrhagic event	0 (0.0)	1 (0.8)	2.60 (0.04-162.04)	NR	1.000	0.720
Systemic embolic event	0 (0.0)	0 (0.0)	NA	NA	NA	NA
Myocardial infarction	0 (0.0)	0 (0.0)	NA	NA	NA	NA
Unplanned urgent revascularization	0 (0.0)	0 (0.0)	NA	NA	NA	NA
Stent thrombosis	0 (0.0)	0 (0.0)	NA	NA	NA	NA
Composite of major ischemic events	1 (1.0)	4 (3.2)	1.98 (0.36-10.82)	2.86 (0.31-26.78)	0.357	0.442
Composite of any ischemic events	1 (1.0)	4 (3.2)	1.90 (0.37-9.82)	2.86 (0.31-26.78)	0.357	0.767
Safety outcomes						
Major bleeding or clinically relevant nonmajor bleeding	12 (11.2)	7 (6.3)	0.64 (0.27-1.50)	0.54 (0.22-1.34)	0.183	0.081
Fatal bleeding	0 (0.0)	0 (0.0)	NA	NA	NA	NA
Major bleeding	5 (4.7)	3 (2.4)	0.60 (0.15-2.42)	0.34 (0.07-1.67)	0.184	0.316
Clinically relevant nonmajor bleeding	7 (6.5)	4 (3.2)	0.73 (0.25-2.11)	0.68 (0.22-2.11)	0.507	0.153
Any bleeding	17 (16.0)	13 (11.3)	0.66 (0.34-1.28)	0.63 (0.32-1.25)	0.183	0.271
Intracranial hemorrhage	0 (0.0)	1 (0.8)	2.58 (0.06-113.67)	NR	1.000	0.432
Gastrointestinal hemorrhage	3 (2.8)	2 (1.6)	NR	1.01 (0.22-4.76)	0.989	NA
Men	n = 406	n = 396				
Primary outcome						
Net adverse clinical events	66 (17.4)	23 (6.0)	0.37 (0.23-0.58)	0.36 (0.23-0.57)	<0.001	
Secondary outcomes						
Efficacy outcomes						
Death	3 (0.9)	1 (0.3)	0.89 (0.14-5.73)	0.73 (0.11-4.59)	0.733	
Cardiovascular cause	2 (0.6)	1 (0.3)	0.45 (0.01-26.37)	NR	1.000	
Noncardiovascular cause	1 (0.3)	0 (0.0)	1.09 (0.13-9.35)	NR	1.000	
Stroke	3 (0.8)	4 (1.1)	1.60 (0.38-6.82)	1.70 (0.40-7.29)	0.475	
Ischemic event	2 (0.5)	3 (0.8)	1.83 (0.33-10.12)	NR	0.982	
Hemorrhagic event	1 (0.2)	1 (0.3)	1.03 (0.06-19.11)	NR	1.000	
Systemic embolic event	0 (0.0)	0 (0.0)	NA	NA	NA	
Myocardial infarction	2 (0.6)	0 (0.0)	NA	NA	NA	
Unplanned urgent revascularization	6 (1.7)	7 (1.8)	1.04 (0.35-3.06)	1.10 (0.38-3.16)	0.861	
Stent thrombosis	0 (0.0)	0 (0.0)	NA	NA	NA	
Composite of major ischemic events	7 (2.0)	4 (1.1)	0.96 (0.32-2.94)	0.88 (0.29-2.64)	0.819	
Composite of any ischemic events	10 (2.9)	11 (2.9)	1.29 (0.57-2.94)	1.25 (0.56-2.80)	0.591	
Safety outcomes						
Major bleeding or clinically relevant nonmajor bleeding	58 (15.1)	16 (4.2)	0.27 (0.16-0.47)	0.27 (0.16-0.47)	<0.001	
Fatal bleeding	0 (0.0)	0 (0.0)	NA	NA	NA	
Major bleeding	17 (4.4)	3 (0.8)	0.24 (0.08-0.71)	0.20 (0.06-0.61)	0.005	
Clinically relevant nonmajor bleeding	45 (11.7)	14 (3.6)	0.30 (0.16-0.71)	0.30 (0.16-0.54)	<0.001	
Any bleeding	82 (21.2)	36 (9.5)	0.43 (0.30-0.63)	0.44 (0.30-0.65)	<0.001	
Intracranial hemorrhage	3 (0.7)	1 (0.3)	0.44 (0.05-4.22)	0.36 (0.04-3.51)	0.381	
Gastrointestinal hemorrhage	10 (2.6)	6 (1.6)	0.71 (0.27-1.89)	0.70 (0.26-1.85)	0.467	

Values are n (%) unless otherwise indicated. Kaplan-Meier survival curves and Cox proportional hazards models were used to compare the effects of edoxaban monotherapy and dual antithrombotic therapy. The multivariable Cox regression models were adjusted for age, body mass index, CHA_2_DS_2_-VASc score, HAS-BLED score, prior revascularization history, and edoxaban dose-reduction criteria, with stratification by sex to assess treatment effects across men and women.

NA = not available; NR = not reported.

In both women and men, the cumulative incidence of any ischemic event and major ischemic event at 12 months did not differ significantly between the edoxaban monotherapy and dual antithrombotic therapy groups. Among women, the estimated cumulative incidence of major bleeding or clinically relevant nonmajor bleeding at 12 months tended to be lower in the edoxaban monotherapy group than in the dual antithrombotic therapy group (6.3% vs 11.2%; adjusted HR: 0.54; 95% CI: 0.22-1.34; *P* = 0.183). In men, the rate of major bleeding or clinically relevant nonmajor bleeding was significantly lower in the edoxaban monotherapy group than in the dual antithrombotic therapy group (4.2% vs 15.1%; adjusted HR: 0.27; 95% CI: 0.16-0.47; *P* < 0.001) (Figure 3). No significant interaction was observed between the randomized antithrombotic strategy and sex category with respect to any ischemic event (*P* for interaction = 0.767), major ischemic event (*P* for interaction = 0.442), and major bleeding or clinically relevant nonmajor bleeding (*P* for interaction = 0.081).

The forest plot for sex and treatment strategies in terms of all clinical outcomes is illustrated in Supplemental Figures 1 and 2. Also, among both women and men, the treatment effect of edoxaban monotherapy vs dual antithrombotic therapy on the primary outcome appeared consistent across all prespecified subgroups (Supplemental Figures 3 and 4).

## Discussion

This prespecified subanalysis of the EPIC-CAD trial evaluated the comparative outcomes of different antithrombotic strategies (edoxaban monotherapy vs dual antithrombotic therapy) stratified by participant sex. The key findings can be summarized as follows (Central Illustration): 1) women were older, had lower body weight and creatinine clearance, higher CHA_2_DS_2_-VASc scores, and a higher proportion of meeting the criteria for edoxaban dose-reduction; 2) the 12-month incidence of primary net adverse events was significantly lower with edoxaban monotherapy than with dual antithrombotic therapy in men, but not in women; 3) the primary and secondary clinical outcomes at 12 months did not differ significantly between women and men and, similarly, the rates of any ischemic or major ischemic events did not differ significantly between edoxaban monotherapy and dual antithrombotic therapy in either sex; and 4) the incidence of clinically relevant bleeding events was significantly lower with edoxaban monotherapy in men, but not in women.

**Central Illustration:**
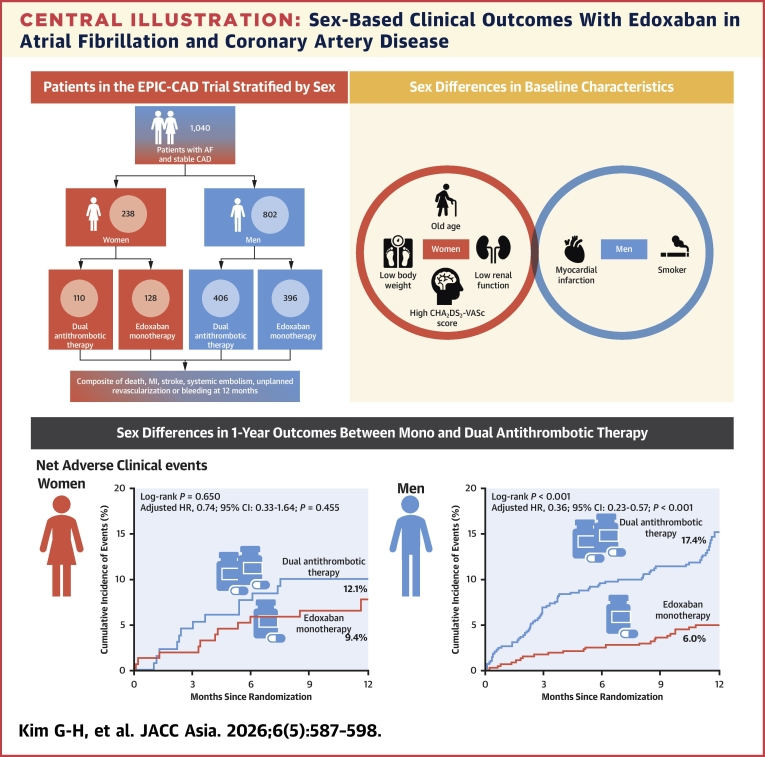
Sex-Based Clinical Outcomes With Edoxaban in Atrial Fibrillation and Coronary Artery Disease This figure shows the sex-specific breakdown and differences in characteristics of the EPIC-CAD trial. In men, there is a significant difference in primary net adverse clinical events between dual therapy and monotherapy. CAD = coronary artery disease; EPIC-CAD = Edoxaban Versus Edoxaban With Antiplatelet Agent in Patients With Atrial Fibrillation and Chronic Stable Coronary Artery Disease Trial; MI = myocardial infarction.

Several reports have suggested sex-related differences in the efficacy and safety of OACs for AF and antiplatelet regimens for CAD. In patients with AF receiving OACs, women have a higher risk of stroke and mortality.^2^ Among women, direct oral anticoagulant (DOAC) use has been associated with a lower risk of intracranial hemorrhage and all-cause mortality compared with warfarin.^3^ Although DOAC therapy for stroke prevention demonstrates similar efficacy in both sexes, it is associated with significantly lower rates of major bleeding in women.^16^ In patients with CAD receiving antiplatelet therapy, the efficacy of antiplatelet therapy was comparable between women and men; however, women have a higher bleeding risk.^17^^,^^18^ Although women with AF have a lower prevalence of CAD than men, treatment decisions regarding the combination and duration of antithrombotic therapy remain particularly challenging because of the underrepresentation of women in pivotal clinical trials. Furthermore, current guidelines do not recommend sex-specific antithrombotic approaches, but instead advise that it be applied equally to both sexes.^19^, ^20^, ^21^ Therefore, this prespecified sex-specific analysis of the EPIC-CAD trial provides important clinical insights into the relative efficacy and safety of edoxaban-based antithrombotic therapy in high-risk patients with concomitant AF and CAD.

Considering the combined use of antiplatelet and anticoagulant regimens in patients with AF and CAD, along with sex differences, antithrombotic regimens for these patients present additional challenges in clinical practice. Prior landmark trials have demonstrated that OAC monotherapy reduces complications while maintaining therapeutic efficacy comparable to that of dual antithrombotic therapy.^14^^,^^22^^,^^23^ The AFIRE (Atrial Fibrillation and Ischemic Events Associated with Rivaroxaban in Patients with Stable Coronary Artery Disease) trial demonstrated that rivaroxaban monotherapy was noninferior in efficacy and superior in safety compared with combination therapy with rivaroxaban and a single antiplatelet agent.^23^ In the sex-specific subgroup analysis of the AFIRE trial, the effect of rivaroxaban monotherapy over combination therapy on the primary efficacy and safety endpoints was more pronounced in men than in women. Specifically, for the primary efficacy endpoint, the HR was 0.69 (95% CI: 0.50-0.93) in men vs 0.90 (95% CI: 0.51-1.58) in women. Similarly, for the primary safety endpoint, the HR was 0.44 (95% CI: 0.27-0.72) in men vs 1.66 (95% CI: 0.66-4.23) in women. These findings align with the key findings of the sex-specific analysis of the EPIC-CAD trial. In the current study, the clinical superiority of edoxaban monotherapy over dual antithrombotic therapy in patients with AF and stable CAD with regard to primary net adverse events and clinically relevant bleeding events was more pronounced in men than in women.

The exact reasons for a lower treatment effect of DOAC monotherapy compared with dual antithrombotic therapy (DOAC plus a single antiplatelet agent) for women in the AFIRE and EPIC-CAD trials remain unclear. A previous pooled analysis of DOAC trials found that, compared with warfarin, DOAC provided greater protection against stroke or systemic embolism in men while offering better protection against major bleeding in women.^24^ In the sex-specific analysis of the ENGAGE AF-TIMI 48 (Effective Anticoagulation with Factor Xa Next Generation in Atrial Fibrillation-Thrombolysis In Myocardial Infarction 48) trial,^25^ the efficacy of edoxaban compared with warfarin was similar between sexes. However, edoxaban was associated with a lower risk of hemorrhagic stroke and several serious bleeding events in women than in men. Given the differences in study design, cohort selection, and endpoint definitions, direct comparisons between our findings and those of other clinical trials and meta-analyses are challenging. One possible explanation for the reduced efficacy of DOAC monotherapy in women may be the higher proportion of low-dose (30 mg) edoxaban use. In the present study, the proportion of women meeting the criteria for edoxaban dose reduction—because of factors such as lower renal function and lower body weight—was approximately twice that of men. Notably, a subgroup analysis of edoxaban dosing in the ENGAGE AF-TIMI 48 trial found that high-dose edoxaban (60 mg) was significantly associated with an increased risk of bleeding compared with low-dose edoxaban (30 mg).^26^ Given that a substantial proportion of women in the dual antithrombotic therapy group received a low dose of edoxaban, it may have contributed to a lower incidence of bleeding events in this group, thereby diminishing the apparent benefit of edoxaban monotherapy in women. Therefore, the combined effect of edoxaban and antiplatelet drugs, particularly in relation to bleeding events, was more pronounced in men, who received a higher proportion of standard-dose edoxaban compared with women, who received a higher proportion of low-dose edoxaban.

### Study limitations

First, as a subgroup analysis of a prospective randomized controlled trial, the present study was limited by an insufficient sample size and reduced statistical power. Therefore, the interpretation of the findings should be considered provisional, and further prospective studies are needed to validate our findings. Second, randomization in the EPIC-CAD trial was not stratified by sex. Although most baseline characteristics were well balanced between the randomized groups, the potential effect of other unmeasured confounders cannot be excluded. The clinician-assessed risk factors such as frailty, a history of falls, or previous bleeding disorders, which are not fully captured in the current dataset, could introduce bias and complicate the interpretation of the study outcomes. Third, the study’s limited sample size and follow-up duration significantly constrained its ability to assess efficacy outcomes, as reflected by the wide confidence interval for some ischemic outcomes. Fourth, in the comparison between women and men, the propensity-matching procedure may have affected the validity of the randomization and intention-to-treat analyses by affecting the characteristics of the study population. Finally, as the study population was limited to East Asian individuals, the generalizability of our findings to other ethnic groups remains uncertain.

## Conclusions

In this prespecified subgroup analysis of the EPIC-CAD trial, no significant treatment differences or interactions were observed according to sex, despite more frequent application of edoxaban dose-reduction criteria in women. These findings highlight the need for further research focusing on sex differences to optimize antithrombotic strategies for patients with concomitant AF and CAD.

## Funding Support and Author Disclosures

This work was supported by grants from the Cardiovascular Research Foundation (Seoul, Korea), Daiichi-Sankyo, and Daewoong Pharmaceutical (Seoul, Korea). Dr S.-J. Park has received research grants from Daewoong Pharm and the Cardiovascular Research Foundation related to this work, as well as lecture fees from Daiichi-Sankyo, Abbott Vascular, Boston Scientific, Medtronic, and Edwards outside the submitted work. Dr Nam received research grants from Daiichi-Sankyo and Daewoong Pharm. Dr D.-W. Park has received research grants from Daiichi-Sankyo, Daewoong Pharm, and the Cardiovascular Research Foundation related to this work, along with lecture fees from Daiichi-Sankyo, Abbott Vascular, Boston Scientific, Medtronic, and Edwards outside the submitted work. All other authors have reported that they have no relationships relevant to the contents of this paper to disclose.
