# Supplementary material for: Sex-Based Differences in Clinical Outcomes With Edoxaban Therapy: A Prespecified Analysis of the EPIC-CAD Trial
Source: JACC Asia. 2026 Feb 28;6(5):587–98. doi: 10.1016/j.jacasi.2025.12.022 (PMC13153895; doi:10.1016/j.jacasi.2025.12.022)
Supplement: Supplemental Tables 1 and 2 and Supplemental Figures 1-4 [file mmc1.docx]

**SUPPLEMENTAL MATERIAL**

The authors have provided this appendix to offer readers additional information about their work.

**Supplemental Table 1.** Baseline Characteristics of Patients Stratified by Treatment Strategy and Sex.

**Supplemental Table 2.** Primary and Secondary Clinical Outcomes at 12 Months by Sex.

**Supplemental Figure 1.** Forest Plot of Clinical Outcomes According to Antithrombotic Treatment Strategies in Women.

**Supplemental Figure 2.** Forest Plot of Clinical Outcomes According to Antithrombotic Treatment Strategies in Men.

**Supplemental Figure 3.** Key Subgroup Analyses of the Primary Outcome in Women.

**Supplemental Figure 4.** Key Subgroup Analyses of the Primary Outcome in Men.

**Supplemental Table 1. Baseline Characteristics of Patients Stratified by Treatment Strategy and Sex***

|  | **Women (n = 238)** | |  | **Men (n = 802)** | |  |
| --- | --- | --- | --- | --- | --- | --- |
| **Characteristics** | **Edoxaban Monotherapy (n = 128)** | **Dual Antithrombotic Therapy (n = 110)** | ***P* Value** | **Edoxaban Monotherapy (n = 396)** | **Dual Antithrombotic Therapy (n =406)** | ***P* Value** |
| Age, y | 74.9 ± 7.0 | 75.6 ± 7.8 | 0.504 | 70.7 ± 8.0 | 71.6 ± 8.4 | 0.097 |
| Weight, kg | 58.9 ± 9.9 | 60.4 ± 9.5 | 0.255 | 71.6 ± 10.1 | 71.2 ± 10.5 | 0.644 |
| Body-mass index | 25.4 ± 4.1 | 25.6 ± 3.4 | 0.729 | 25.4 ± 3.0 | 25.2 ± 3.3 | 0.533 |
| Cardiac risk factors and comorbidities |  |  |  |  |  |  |
| Diabetes mellitus | 60 (46.9) | 37 (33.6) | 0.052 | 164 (41.4) | 160 (39.4) | 0.612 |
| Hypertension | 105 (82.0) | 90 (81.8) | 1.000 | 318 (80.3) | 332 (81.8) | 0.659 |
| Hyperlipidemia or statin use | 124 (96.9) | 103 (93.6) | 0.381 | 366 (92.4) | 379 (93.3) | 0.709 |
| Current smoker | 2 (1.6) | 4 (3.6) | 0.420 | 35 (8.8) | 46 (11.3) | 0.369 |
| Previous MI | 12 (9.4) | 18 (16.4) | 0.155 | 67 (16.9) | 74 (18.2) | 0.694 |
| Congestive heart failure | 28 (21.9) | 20 (18.2) | 0.585 | 68 (17.2) | 89 (21.9) | 0.108 |
| History of cerebrovascular disease | 22 (17.2) | 20 (18.2) | 0.976 | 55 (13.9) | 57 (14.0) | 1.000 |
| History of peripheral artery disease | 5 (3.9) | 10 (9.1) | 0.170 | 28 (7.1) | 35 (8.6) | 0.494 |
| Creatinine clearance, ml/min | 57.2 ± 19.7 | 58.2 ± 23.0 | 0.722 | 70.2 ± 23.9 | 68.1 ± 20.5 | 0.187 |
| Type of atrial fibrillation |  |  | 0.801 |  |  | 0.703 |
| Paroxysmal | 76 (59.4) | 68 (61.8) |  | 216 (54.5) | 215 (53.0) |  |
| Persistent or permanent | 52 (40.6) | 42 (38.2) |  | 180 (45.5) | 191 (47.0) |  |
| CHA_2_DS_2_-VASc score |  |  |  |  |  |  |
| Mean | 5.3 ± 1.5 | 5.2 ± 1.6 | 0.442 | 4.0 ± 1.4 | 4.1 ± 1.4 | 0.121 |
| Median (IQR) | 5 (4-6) | 5 (4-6) |  | 4 (3-5) | 4 (3-5) |  |
| CHA_2_DS_2_ score |  |  |  |  |  |  |
| Mean | 2.4 ± 1.3 | 2.3 ± 1.3 | 0.479 | 2.0 ± 1.1 | 2.1 ± 1.2 | 0.193 |
| Median (IQR) | 2 (2-3) | 2 (2-3) |  | 2 (1-3) | 2 (1-3) |  |
| HAS-BLED score |  |  |  |  |  |  |
| Mean | 2.2 ± 0.8 | 2.3 ± 0.8 | 0.267 | 2.1 ± 0.8 | 2.1 ± 0.9 | 0.779 |
| Median (IQR) | 2 (2-3) | 2 (2-3) |  | 2 (2-3) | 2 (2-3) |  |
| Obstructive CAD managed medically | 52 (40.6) | 41 (37.3) | 0.484 | 136 (34.3) | 128 (31.5) | 0.515 |
| Previous coronary revascularization | 76 (59.4) | 69 (62.7) | 0.693 | 260 (65.7) | 278 (68.5) | 0.439 |
| Previous PCI | 67 (52.3) | 64 (58.2) | 0.440 | 241 (60.9) | 254 (62.6) | 0.672 |
| Drug-eluting stent, no./total no. (%) | 54 (80.6) | 56 (87.5) | 0.402 | 197 (81.7) | 211 (83.1) | 0.787 |
| Bare-metal stent, no./total no. (%) | 2 (3.0) | 1 (1.6) | 1.000 | 11 (4.6) | 6 (2.4) | 0.272 |
| Both stent type, no./total no. (%) | 2 (3.0) | 0 (0.0) | 0.496 | 6 (2.5) | 4 (1.6) | 0.687 |
| Unknown stent type, no./total no. (%) | 9 (13.4) | 7 (10.9) | 0.866 | 27 (11.2) | 33 (13.0) | 0.637 |
| Previous CABG | 15 (11.7) | 6 (5.5) | 0.142 | 26 (6.6) | 30 (7.4) | 0.750 |
| Previous rhythm control strategy |  |  |  |  |  |  |
| Previous RFCA | 38 (29.7) | 32 (29.1) | 1.000 | 99 (25.0) | 113 (27.8) | 0.407 |
| Previous or concomitant PPI use | 14 (10.9) | 17 (15.5) | 0.401 | 45 (11.4) | 57 (14.0) | 0.302 |
| Indication for dose adjustment of edoxaban | 84 (65.6) | 63 (57.3) | 0.235 | 94 (23.7) | 105 (25.9) | 0.539 |
| Edoxaban dose |  |  | 1.000 |  |  | 0.009 |
| 60mg/day | 43 (33.6) | 37 (33.6) |  | 274 (69.2) | 244 (60.1) |  |
| 30mg/day | 85 (66.4) | 73 (66.4) |  | 122 (30.8) | 162 (39.9) |  |
| Antiplatelet drug used |  |  | <0.001 |  |  | <0.001 |
| Aspirin | 0 (0.0) | 76 (69.1) |  | 1 (0.3) | 243 (59.9) |  |
| Clopidogrel | 0 (0.0) | 34 (30.9) |  | 2 (0.5) | 161 (39.7) |  |

*Values are presented as mean ± standard deviation or n (%) unless otherwise stated. Percentages may not sum to 100% due to rounding. CABG indicates coronary artery bypass grafting; CAD, coronary artery disease; MI, myocardial infarction; PCI, percutaneous coronary intervention; PPI, proton pump inhibitor; and RFCA, radiofrequency catheter ablation.

**Supplemental Table 2. Primary and Secondary Clinical Outcomes at 12 Months between Women and Men.**

| **Outcomes** | **No. (%)** | | | | |  | **HR (95% CI)** | | | |  | |  |
| --- | --- | --- | --- | --- | --- | --- | --- | --- | --- | --- | --- | --- | --- |
|  | **Women**  **(n = 238)** | | **Men**  **(n = 802)** |  | **Unadjusted** | | | | **Adjusted*** | | ***P* Value*** | |  |
| **Primary outcome** |  | |  |  |  | | | |  | |  | |  |
| Net adverse clinical events | 24 (10.6) | | 89 (11.8) |  | 1.08 (0.70-1.65) | | | | 1.15 (0.71-1.87) | | 0.559 | |  |
| **Secondary Outcomes** |  | |  |  |  | | | |  | |  | |  |
| Efficacy outcomes |  | |  |  |  | | | |  | |  | |  |
| Death | 2 (0.8) | | 4 (0.5) |  | 0.61 (0.14-2.62) | | | | 1.37 (0.26-7.06) | | 0.709 | |  |
| Cardiovascular cause | 0 (0.0) | | 3 (0.3) |  | 0.22 (0.02-2.57) | | | | NR | | NR | |  |
| Noncardiovascular cause | 2 (0.8) | | 1 (0.1) |  | NA | | | | NA | | NA | |  |
| Stroke | 4 (1.7) | | 7 (0.9) |  | 0.59 (0.18-1.97) | | | | 0.34 (0.08-1.40) | | 0.136 | |  |
| Ischemic event | 3 (1.2) | | 5 (0.6) |  | 0.20 (0.03-1.23) | | | | NR | | NR | |  |
| Hemorrhagic event | 1 (0.4) | | 2 (0.2) |  | 0.66 (0.06-7.54) | | | | NR | | NR | |  |
| Systemic embolic event | 0 (0.0) | | 0 (0.0) |  | NA | | | | NA | | NA | |  |
| Myocardial infarction | 0 (0.0) | | 2 (0.2) |  | NA | | | | NA | | NA | |  |
| Unplanned urgent revascularization | 0 (0.0) | | 13 (1.7) |  | NA | | | | NA | | NA | |  |
| Stent thrombosis | 0 (0.0) | | 0 (0.0) |  | NA | | | | NA | | NA | |  |
| Composite of major ischemic events | 5 (2.1) | | 11 (1.5) |  | 0.68 (0.26-1.78) | | | | 0.83 (0.27-2.58) | | 0.752 | |  |
| Composite of any ischemic events | 5 (2.1) | | 21 (2.6) |  | 1.27 (0.52-3.14) | | | | 1.48 (0.53-4.07) | | 0.453 | |  |
| Safety outcomes |  | |  |  |  | | |  | | | |  |  |
| Major bleeding or clinically relevant nonmajor bleeding | 19 (8.2) | 74 (9.5) | | | |  | 1.09 (0.68-1.77) | 1.15 (0.66-1.97) | | 0.625 | | |  |
| Fatal bleeding | 0 (0.0) | 0 (0.0) | | | |  | NA | NA | | NA | | |  |
| Major bleeding | 8 (3.4) | 20 (2.5) | | | |  | 0.81 (0.36-1.83) | 0.99 (0.40-2.48) | | 0.985 | | |  |
| Clinically relevant nonmajor bleeding | 11 (4.7) | 59 (7.5) | | | |  | 1.42 (0.78-2.58) | 1.36 (0.70-2.64) | | 0.370 | | |  |
| Any bleeding | 29 (12.5) | 118 (15.1) | | | |  | 1.05 (0.73-1.52) | 1.01 (0.66-1.54) | | 0.963 | | |  |
| Intracranial hemorrhage | 1 (0.4) | 4 (0.5) | | | |  | 1.18 (0.13-10.53) | 1.79 (0.16-19.52) | | 0.635 | | |  |
| Gastrointestinal hemorrhage | 5 (2.1) | 11 (1.5) | | | |  | 0.73 (0.30-1.77) | 0.71 (0.26-1.96) | | 0.510 | | |  |

*Kaplan-Meier survival curves and Cox proportional hazards models were used to compare treatment effects between women and men. Samples were matched using propensity score based on age, BMI, hypertension (HTN), diabetes mellitus (DM), history of cerebrovascular accident (CVA), CHA_2_DS_2_-VASc score, HAS-BLED score, prior revascularization history, and the presence of edoxaban dose reduction criteria. The multivariable Cox regression models were adjusted for age, BMI, CHA_2_DS_2_-VASc score, HAS-BLED score, prior revascularization history, and edoxaban dose-reduction criteria to assess treatment effects in both men and women. NA indicates not available; and NR, not reported.**
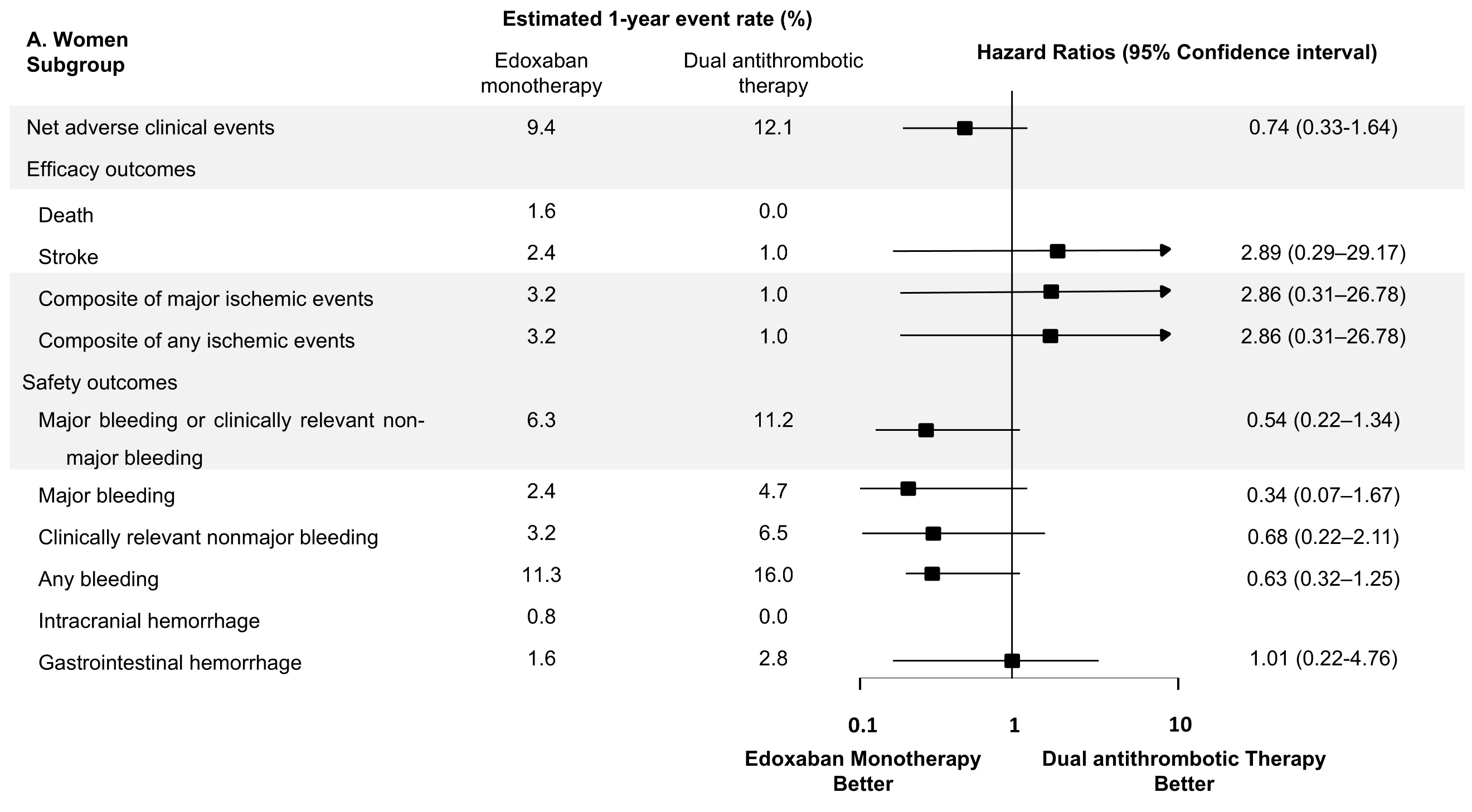
Supplemental Figure 1. Forest Plot of Clinical Outcomes According to Antithrombotic Treatment Strategies in Women**

**
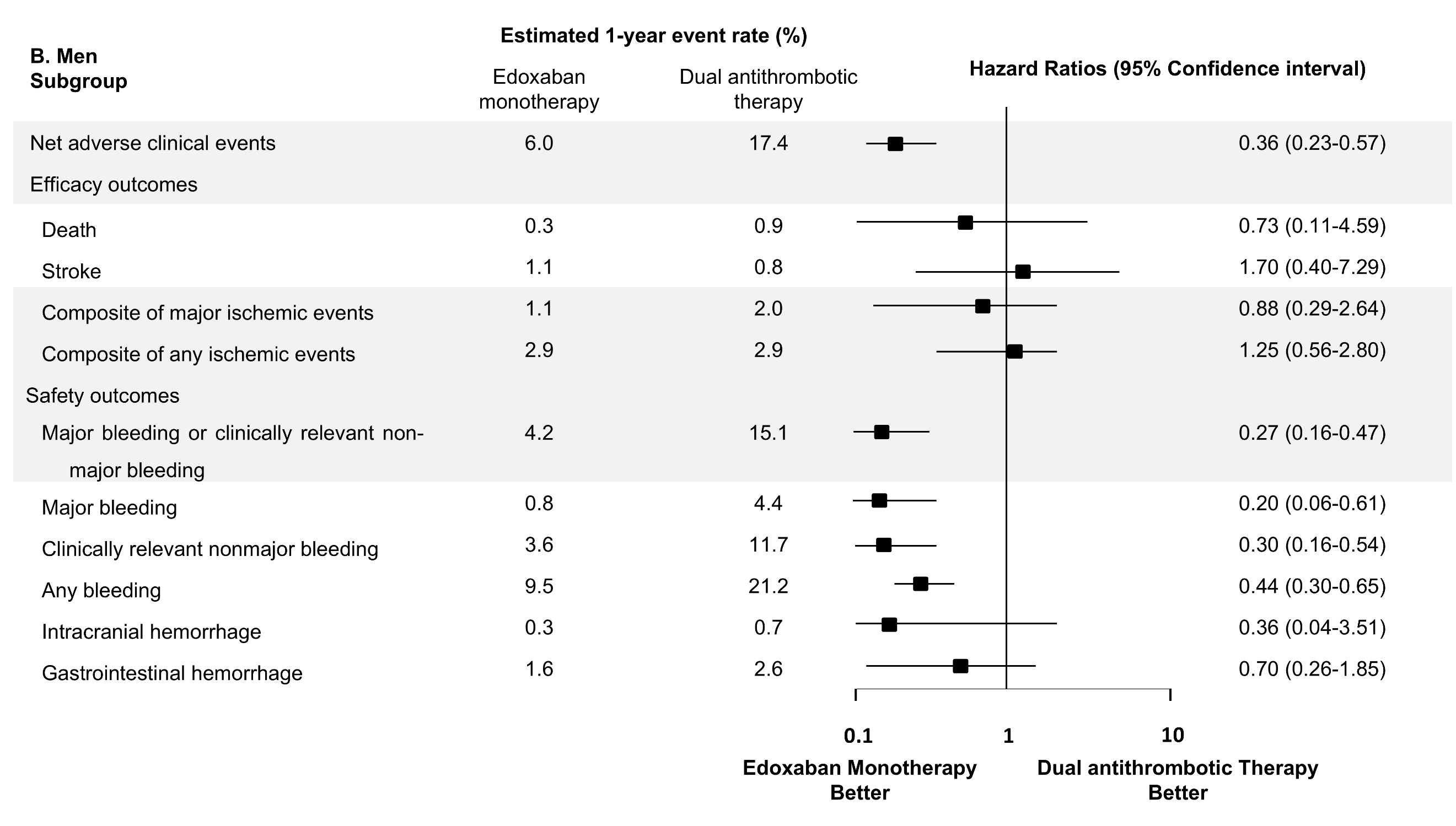
Supplemental Figure 2. Forest Plot of Clinical Outcomes According to Antithrombotic Treatment Strategies in Men**

**
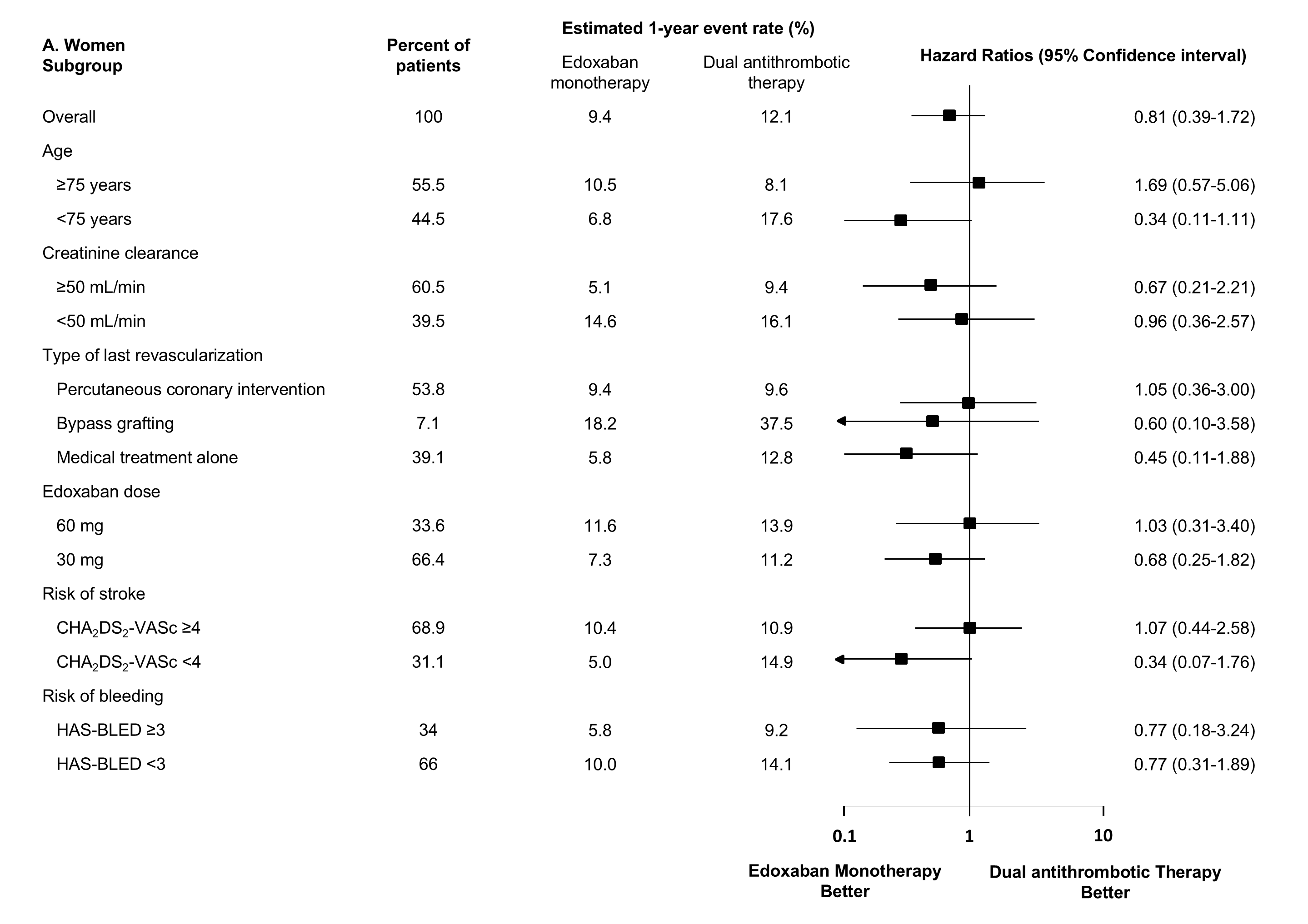
Supplemental Figure 3. Key Subgroup Analyses of the Primary Outcome in Women**

**
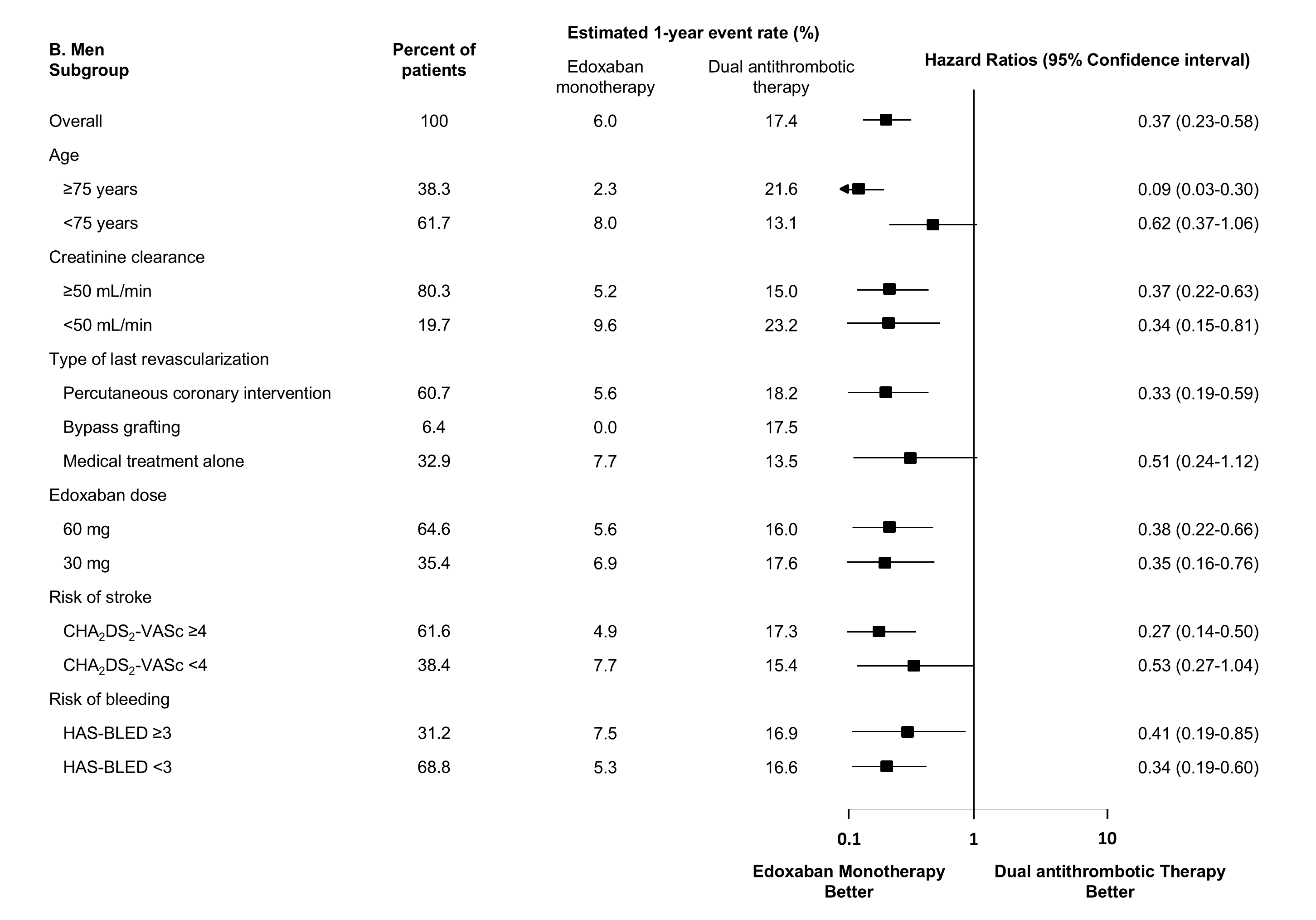
Supplemental Figure 4. Key Subgroup Analyses of the Primary Outcome in Men**
